# Author Correction: Host-derived protein profiles of human neonatal meconium across gestational ages

**DOI:** 10.1038/s41467-025-61278-z

**Published:** 2025-06-25

**Authors:** Yoshihiko Shitara, Ryo Konno, Masahito Yoshihara, Kohei Kashima, Atsushi Ito, Takeo Mukai, Goh Kimoto, Satsuki Kakiuchi, Masaki Ishikawa, Tomo Kakihara, Takeshi Nagamatsu, Naoto Takahashi, Jun Fujishiro, Eiryo Kawakami, Osamu Ohara, Yusuke Kawashima, Eiichiro Watanabe

**Affiliations:** 1 Department of Pediatrics, Faculty of Medicine, The University of Tokyo, Tokyo, Japan; 2 Department of Applied Genomics, Kazusa DNA Research Institute, Chiba, Japan; 3 Institute for Advanced Academic Research (IAAR), Chiba University, Chiba, Japan; 4 Department of Artificial Intelligence Medicine, Graduate School of Medicine, Chiba University, Chiba, Japan; 5 Premium Research Institute for Human Metaverse Medicine (WPI-PRIMe), Osaka University, Suita, Osaka Japan; 6 Department of Pediatric Surgery, Faculty of Medicine, The University of Tokyo, Tokyo, Japan; 7 Department of Obstetrics and Gynecology, Faculty of Medicine, International University of Health and Welfare, Chiba, Japan; 8 Advanced Data Science Project, RIKEN Information R&D and Strategy Headquarters, RIKEN, Kanagawa, Japan; 9 Department of Surgery, Gunma Children’s Medical Center, Gunma, Japan

**Keywords:** Proteomics, Intrauterine growth, Paediatrics

Correction to: *Nature Communications* 10.1038/s41467-024-49805-w, published online 17 July 2024

In the version of the article initially published, Fig. 4f was inadvertently a duplicate of Fig. 4e. The figure has now been amended in the HTML and PDF versions of the article with the correct graphs in Fig. 4f.

