# Supplementary material for: Host-derived protein profiles of human neonatal meconium across gestational ages
Source: Nat Commun. 2024 Jul 17;15:5543. doi: 10.1038/s41467-024-49805-w (PMC11255260; doi:10.1038/s41467-024-49805-w)
Supplement: Supplementary file 1 — Supplementary Information [file 41467_2024_49805_MOESM1_ESM.pdf]

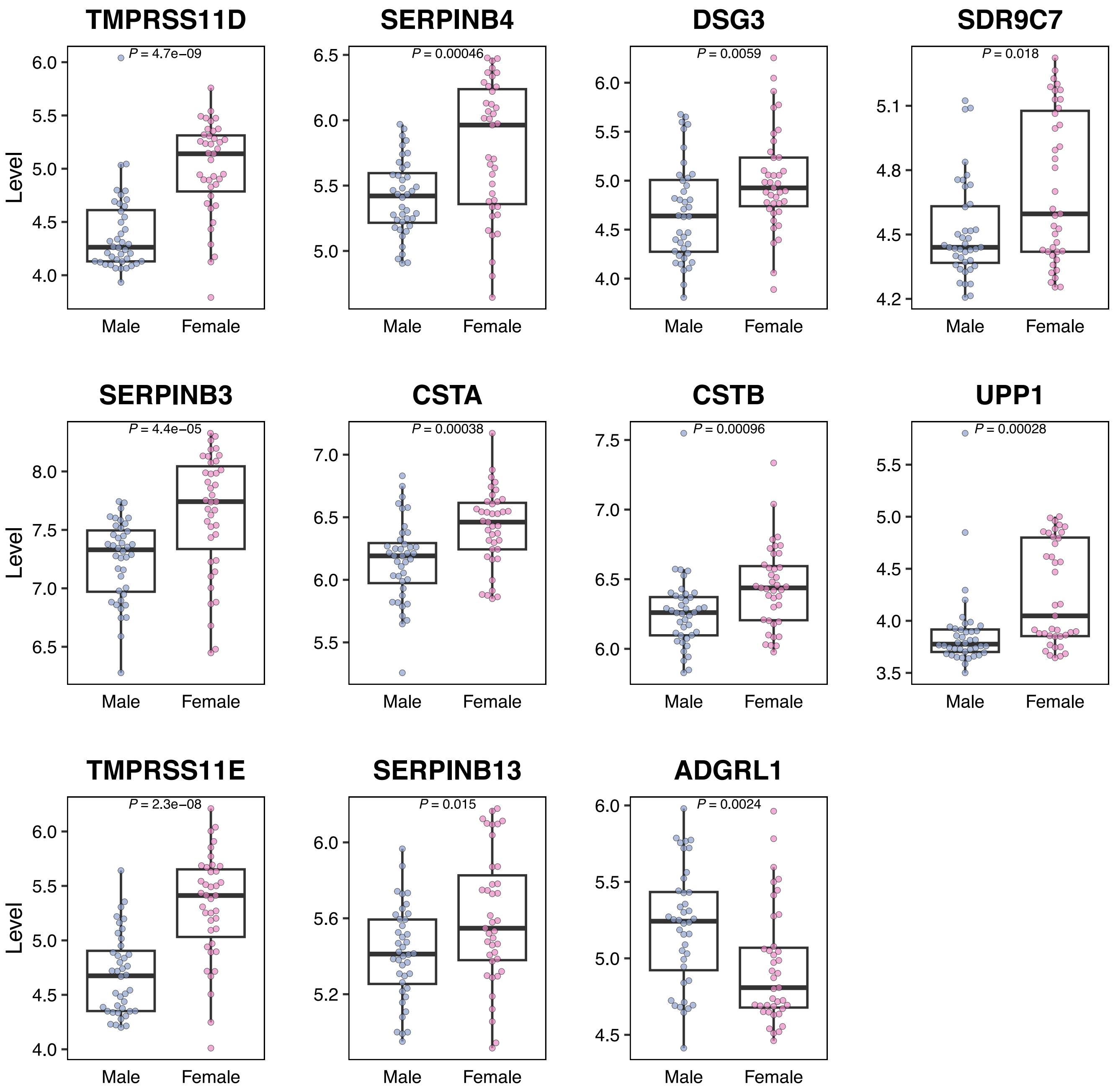

Supplementary Figure 1. Protein abundance in females or males in an independent cohort comprising 79 non-diseased newborns.

Blue and red dots represent males and females, respectively. The centrelines within the box plots represent the medians. The box limits indicate the 25th and 75th percentiles, and the whiskers extend to 1.5 times the interquartile range of the 25th and 75th percentiles. Statistical analyses were performed using the two-sided Wilcoxon rank-sum test.

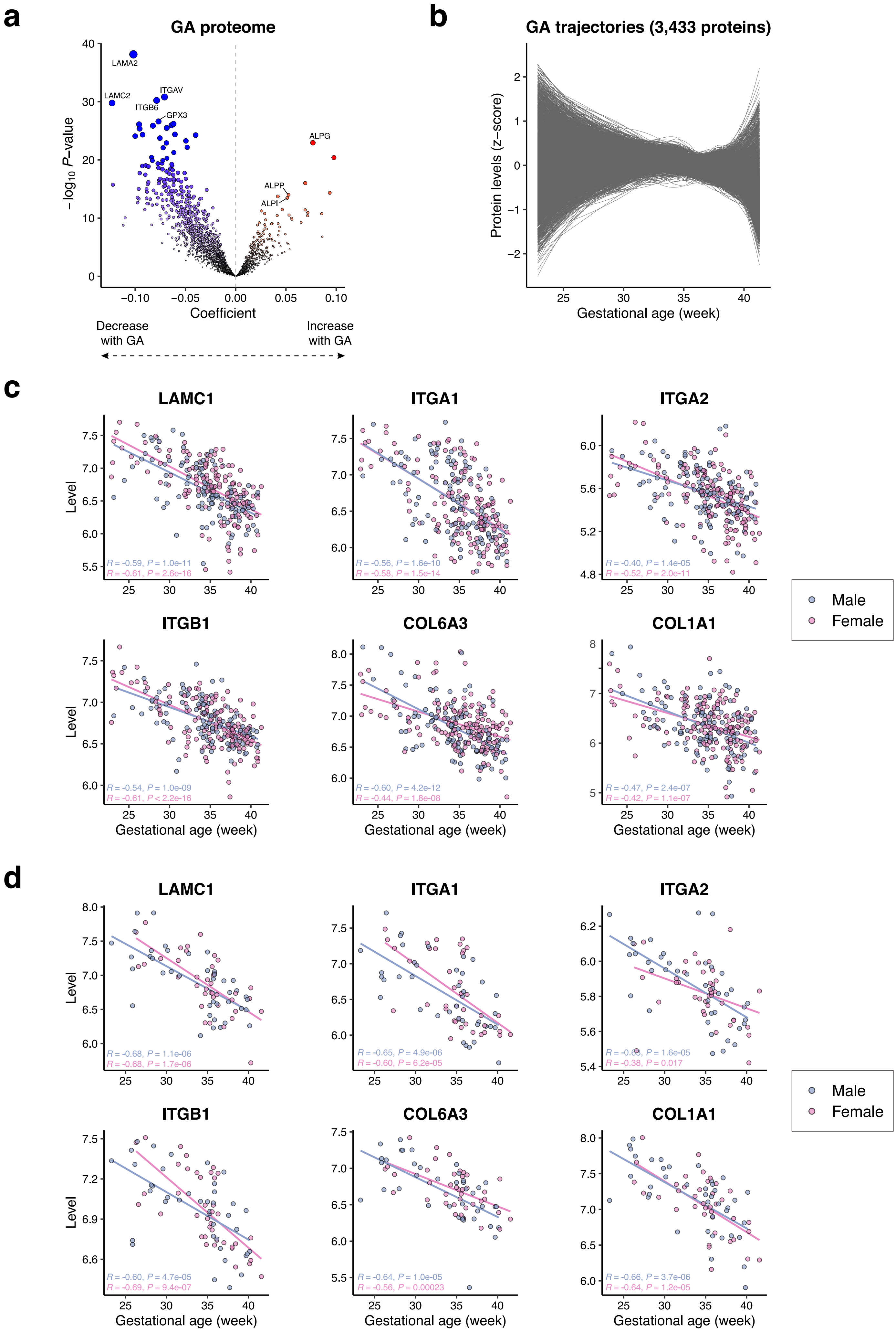

a
GA proteome
LAMA2
LAMC2
ITGAV
ITGB6
GPX3
ALPG
ALPP
ALPI
−log10 P−value
Coefficient
Decrease with GA
Increase with GA
b
GA trajectories (3,433 proteins)
Protein levels (z−score)
Gestational age (week)
c
LAMC1
R = -0.59, P = 1.0e-11
R = -0.61, P = 2.6e-16
ITGA1
R = -0.56, P = 1.6e-10
R = -0.58, P = 1.5e-14
ITGA2
R = -0.40, P = 1.4e-05
R = -0.52, P = 2.0e-11
ITGB1
R = -0.54, P = 1.0e-09
R = -0.61, P < 2.2e-16
COL6A3
R = -0.60, P = 4.2e-12
R = -0.44, P = 1.8e-08
COL1A1
R = -0.47, P = 2.4e-07
R = -0.42, P = 1.1e-07
Level
Gestational age (week)
Male
Female
d
LAMC1
R = -0.68, P = 1.1e-06
R = -0.68, P = 1.7e-06
ITGA1
R = -0.65, P = 4.9e-06
R = -0.60, P = 6.2e-05
ITGA2
R = -0.68, P = 1.6e-05
R = -0.38, P = 0.017
ITGB1
R = -0.60, P = 4.7e-05
R = -0.69, P = 9.4e-07
COL6A3
R = -0.64, P = 1.0e-05
R = -0.56, P = 0.00023
COL1A1
R = -0.66, P = 3.7e-06
R = -0.64, P = 1.2e-05
Level
Gestational age (week)
Male
Female

Supplementary Figure 2. Host-derived meconium proteome during gestational ageing.

a, Volcano plots showing the changes in the meconium proteome with GA (gestational age). The x-axis represents the effect size, indicated by the coefficient, while the y-axis displays the statistical significance, represented by $-\log_{10}$(*P*-value). Positive coefficients indicate higher abundance in the normal gestation, while negative coefficients indicate higher abundance in the premature gestation. *P*-values for each coefficient were calculated using a two-sided t-test without adjustment for multiple testing. **b,** Protein trajectories during gestational ageing. Protein levels were z-scored, and the trajectories of the 3,433 proteins were estimated by locally estimated scatterplot smoothing (LOESS). Each line represents one protein. **c,** Representative extracellular matrix protein expression changes during gestational ageing in a cohort of 259 samples. **d,** Representative extracellular matrix protein expression changes during gestational ageing in an external cohort of 79 samples. Blue and red dots represent males and females, respectively. Pearson correlation coefficients and two-sided *P*-values between the protein expression levels and GA are shown.

a

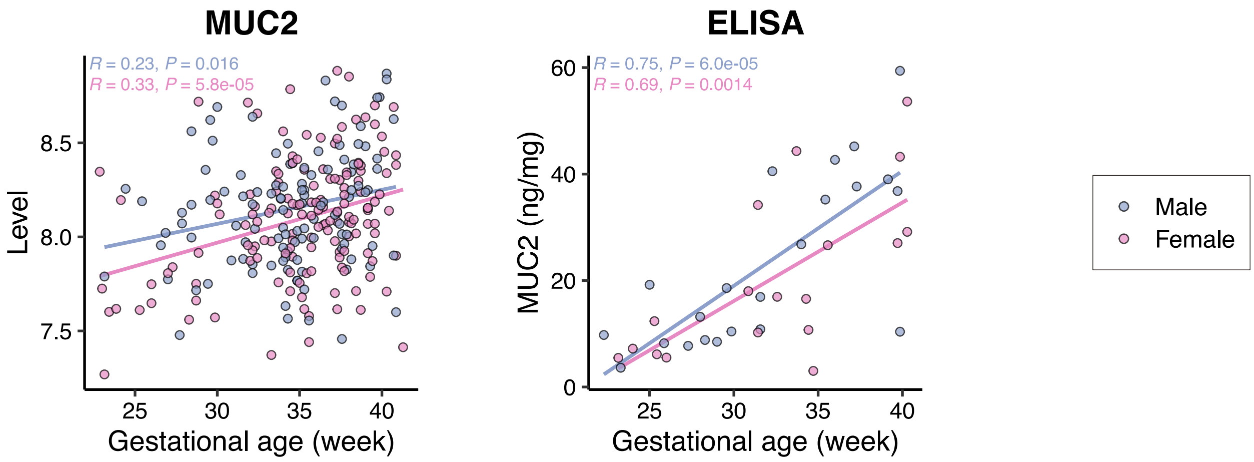

b

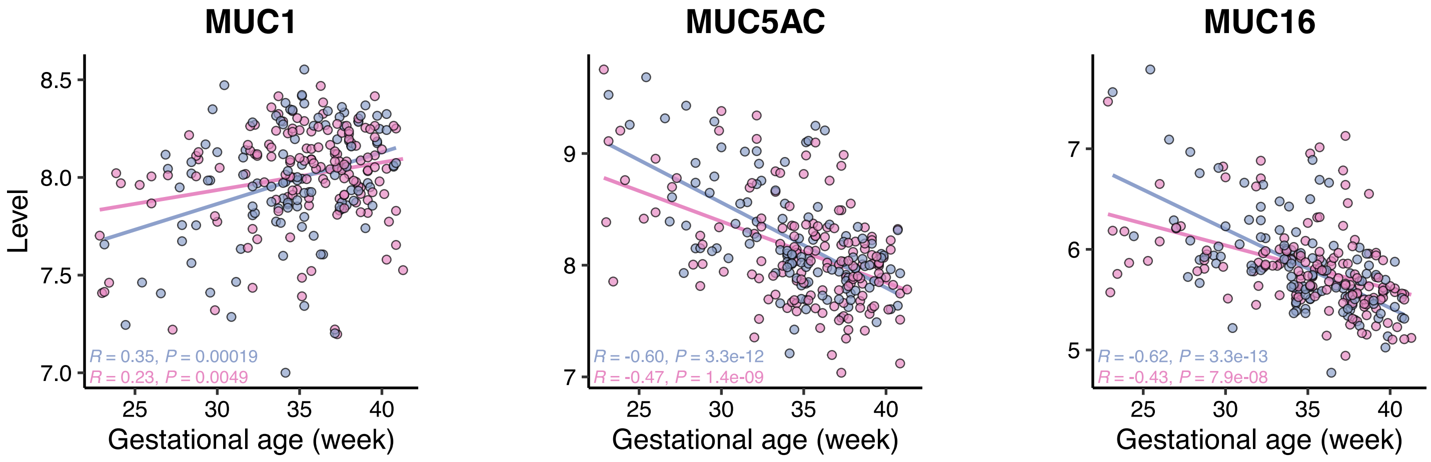

Supplementary Figure 3. Representative mucin protein expression changes during gestational ageing.

a, MUC2 protein expression changes during gestational ageing. Left, Proteome analysis for MUC2 in a cohort of 259 samples. Right, ELISA analysis for human MUC2 protein in another cohort of 40 samples. **b,** MUC1, MUC5AC, and MUC16 protein expression changes during gestational ageing in a cohort comprising 259 samples. Blue and red dots represent males and females, respectively. Pearson correlation coefficients and two-sided *P*-values between the protein expression levels and gestational ages are shown.

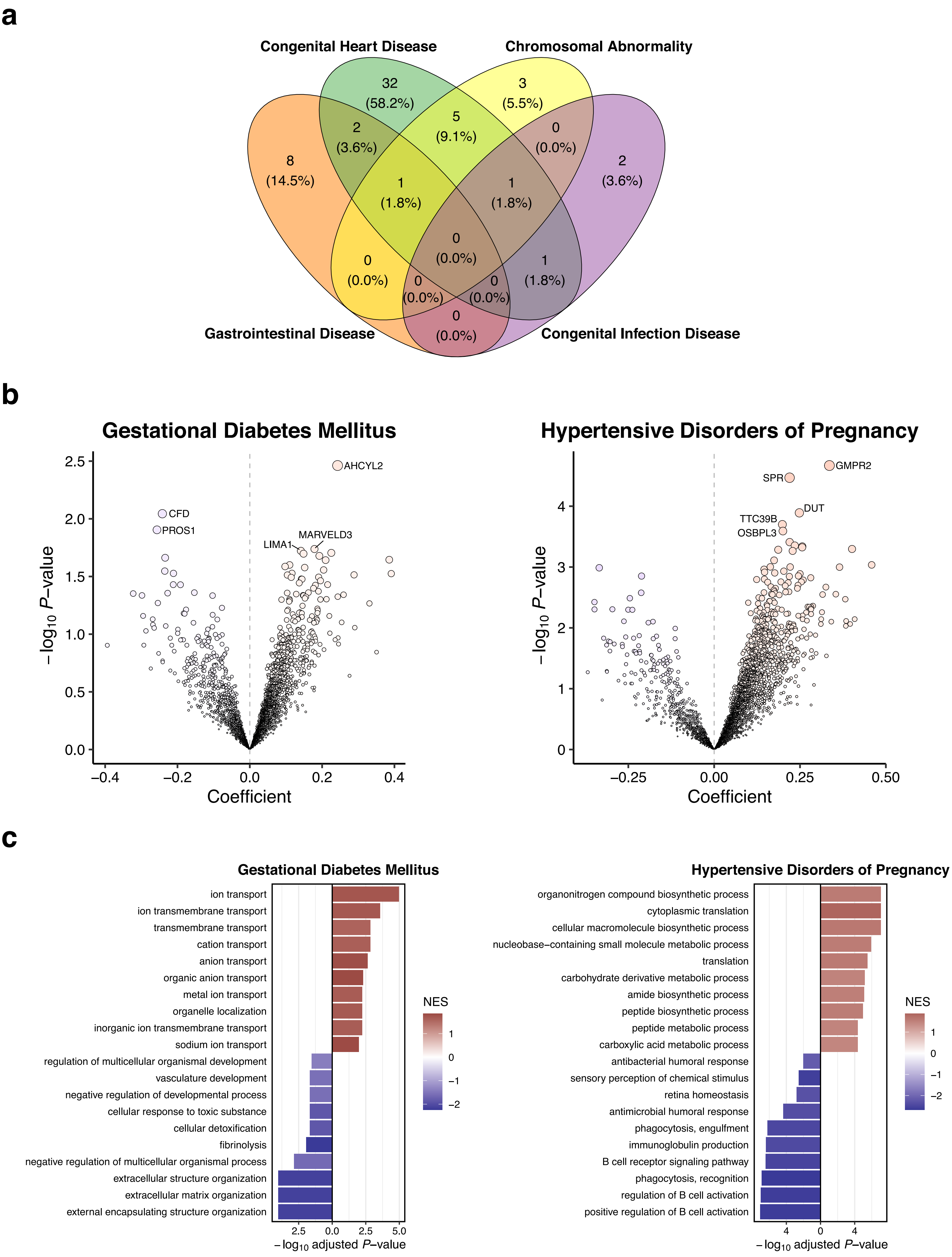

a
Congenital Heart Disease
Chromosomal Abnormality
Gastrointestinal Disease
Congenital Infection Disease
32 (58.2%)
3 (5.5%)
8 (14.5%)
2 (3.6%)
5 (9.1%)
0 (0.0%)
2 (3.6%)
1 (1.8%)
1 (1.8%)
0 (0.0%)
0 (0.0%)
1 (1.8%)
0 (0.0%)
0 (0.0%)
0 (0.0%)
b
Gestational Diabetes Mellitus
AHCYL2
CFD
PROS1
MARVELD3
LIMA1
−log10 P−value
Coefficient
Hypertensive Disorders of Pregnancy
GMPR2
SPR
DUT
TTC39B
OSBPL3
c
Gestational Diabetes Mellitus
ion transport
ion transmembrane transport
transmembrane transport
cation transport
anion transport
organic anion transport
metal ion transport
organelle localization
inorganic ion transmembrane transport
sodium ion transport
regulation of multicellular organismal development
vasculature development
negative regulation of developmental process
cellular response to toxic substance
cellular detoxification
fibrinolysis
negative regulation of multicellular organismal process
extracellular structure organization
extracellular matrix organization
external encapsulating structure organization
NES
−log10 adjusted P−value
Hypertensive Disorders of Pregnancy
organonitrogen compound biosynthetic process
cytoplasmic translation
cellular macromolecule biosynthetic process
nucleobase−containing small molecule metabolic process
translation
carbohydrate derivative metabolic process
amide biosynthetic process
peptide biosynthetic process
peptide metabolic process
carboxylic acid metabolic process
antibacterial humoral response
sensory perception of chemical stimulus
retina homeostasis
antimicrobial humoral response
phagocytosis, engulfment
immunoglobulin production
B cell receptor signaling pathway
phagocytosis, recognition
regulation of B cell activation
positive regulation of B cell activation
NES
−log10 adjusted P−value

Supplementary Figure 4. Correlation between diseases and host-derived meconium proteome.

a, A Venn diagram showing the overlap of cases between diseases in newborns, including GID, CHD, CA, and CID. **b,** Volcano plots showing the changes in the meconium proteome with gestational diabetes mellitus (left) and hypertensive disorders of pregnancy (right). The x-axis represents the effect size, indicated by the coefficient, while the y-axis displays the statistical significance, represented by $-\log_{10}(P\text{-value})$. Positive coefficients indicate higher abundance in the disease samples, while negative coefficients indicate lower abundance. *P*-values for each coefficient were calculated using a two-sided t-test without adjustment for multiple testing. **c,** Gene set enrichment analysis (GSEA) for maternal diseases. Bar plots showing the top 10 significantly enriched GO terms of proteins that are more abundant in the disease samples (red; gestational diabetes mellitus (left) and hypertensive disorders of pregnancy (right)) and less abundant in the disease samples (blue). *P*-values were calculated by permutation test with Benjamini–Hochberg correction, based on the GSEA algorithm as implemented in the R package fgsea. NES: normalised enrichment score.

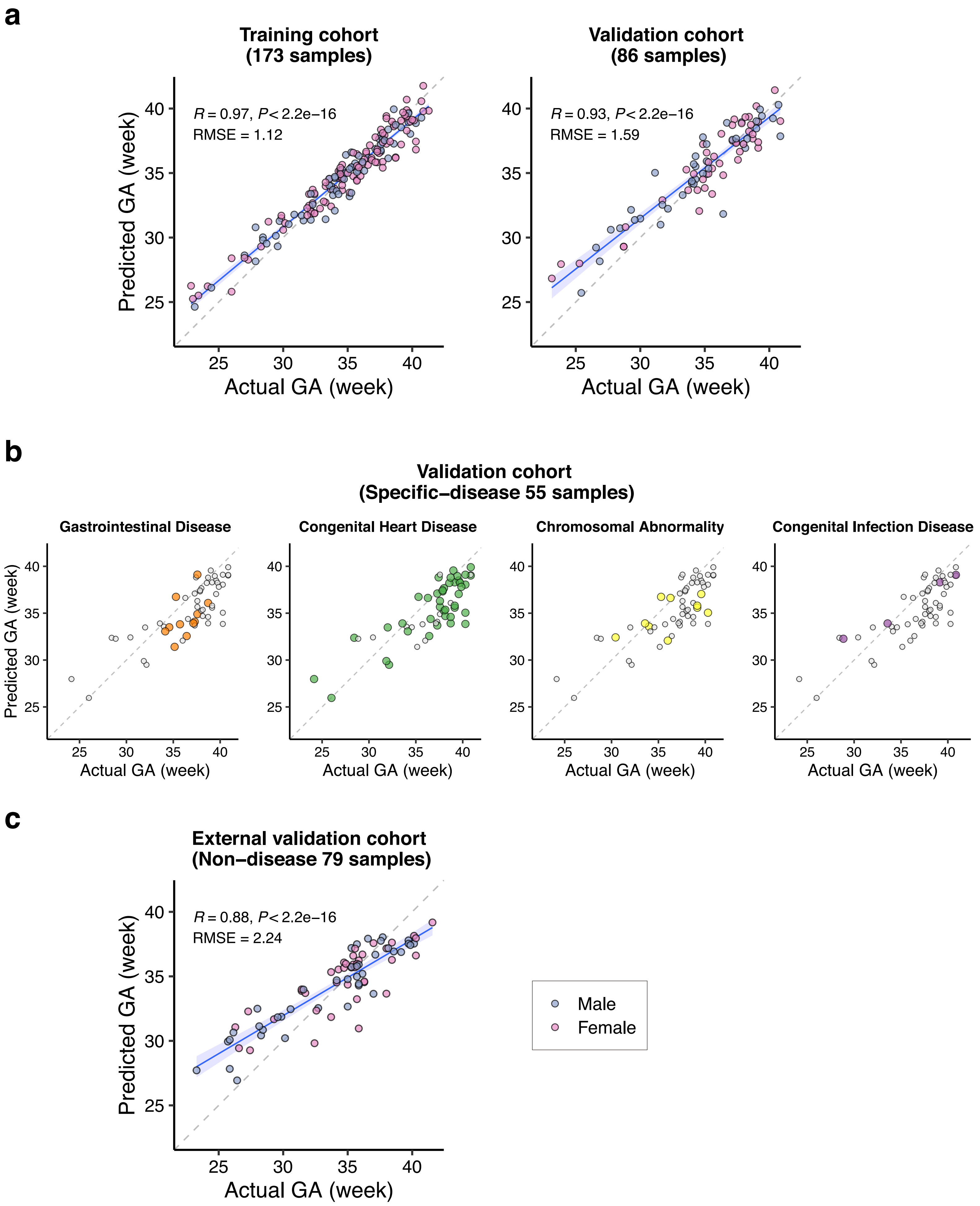
a
Training cohort
(173 samples)
R = 0.97, P < 2.2e−16
RMSE = 1.12
Validation cohort
(86 samples)
R = 0.93, P < 2.2e−16
RMSE = 1.59
Predicted GA (week)
Actual GA (week)
b
Validation cohort
(Specific−disease 55 samples)
Gastrointestinal Disease
Congenital Heart Disease
Chromosomal Abnormality
Congenital Infection Disease
c
External validation cohort
(Non−disease 79 samples)
R = 0.88, P < 2.2e−16
RMSE = 2.24
Male
Female

Supplementary Figure 5. Establishment and validation of gestational age prediction model.

a, Prediction of gestational age (GA) by meconium proteome independent of disease status. Left, GA prediction in the training cohort (173 samples). Right, GA prediction in the validation cohort (86 samples). The training and validation cohorts were randomly assigned at a 2:1 ratio. **b,** GA prediction of the 55 specific-disease samples in the validation cohort. Plots are shown separately for each disease. **c,** GA prediction in the external validation cohort (excluding specific diseases, 79 samples). The blue and red dots represent males and females, respectively. Pearson correlation coefficients and two-sided *P*-values between the actual GA and predicted GA are shown. RMSE: root-mean-square error. The shaded areas around the regression lines represent the 95% confidence interval.

Supplementary Table 1. List of causes for premature birth

Causes of premature birth	N=163
Threatened preterm laber	57
NRFS, Non-reassuring fetal status	38
PROM, Premature rupture of membranes	36
PE, Preeclampsia	24
Previa	12
Growth arrest	10
Placental abruption	7
Intrauterine infection	6
Abnormal blood flow	4
TTTS, Twin-to-twin syndrome	4
CAOS, Chronic abruption-oligohydramnios sequence	3
HDP, Hypertensive disorder of pregnancy	2
Uterine rupture	2
HELLP syndrome	2
Clinical CAM, chorioamnionitis	1
Others	11

Some cases may involve multiple factors.

Supplementary Table 2. Mucin profiles in the initial meconium samples at different gestational ages

Cluster	UniProt Accession Number	Gene Symbol	Protein Name	Coefficient	P-value	adj. P-value
1	P15941	MUC1	Mucin-1	0.01906446	4.36E-06	-1.28E-02
1	Q02817	MUC2	Mucin-2	0.022229963	3.13E-06	6.74E-02
1	Q02505	MUC3A	Mucin-3A	0.025093684	3.94E-05	-1.44E-02
5	Q99102	MUC4	Mucin-4	0.000100667	9.91E-01	-9.20E-02
4	P98088	MUC5AC	Mucin-5AC	-0.063285263	9.56E-20	6.66E-02
6	Q9HC84	MUC5B	Mucin-5B	-0.024002563	3.24E-03	7.97E-02
1	Q6W4X9	MUC6	Mucin-6	0.029348123	2.95E-04	6.84E-02
1	Q8TAX7	MUC7	Mucin-7	0.053615195	2.24E-05	-1.40E-01
6	Q9UKN1	MUC12	Mucin-12	0.014366184	8.37E-02	6.17E-03
6	Q9H3R2	MUC13	Mucin-13	0.007731068	2.74E-01	8.80E-02
4	Q8WXI7	MUC16	Mucin-16	-0.057430953	1.62E-18	-3.74E-03
5	Q685J3	MUC17	Mucin-17	-0.004744885	4.14E-01	2.44E-02

Positive coefficients indicate higher abundance in the normal gestation, while negative coefficients indicate higher abundance in the premature gestation.

P-values for each coefficient were calculated using a two-sided t-test. Adjusted P-values are Benjamini–Hochberg corrected.
