# Supplementary material for: Host-derived protein profiles of human neonatal meconium across gestational ages
Source: Nat Commun. 2024 Jul 17;15:5543. doi: 10.1038/s41467-024-49805-w (PMC11255260; doi:10.1038/s41467-024-49805-w)
Supplement: Supplementary file 3 — Description of Additional Supplementary Files [file 41467_2024_49805_MOESM3_ESM.pdf]

Description of Additional Supplementary Files

File Name: Supplementary Data 1
Description: List of significantly different proteins between males and females.

File Name: Supplementary Data 2
Description: List of clustering for 3,433 proteins.

File Name: Supplementary Data 3
Description: Top 10 significantly enriched gene ontology (GO) terms in each cluster.

File Name: Supplementary Data 4
Description: Clinical characteristics of specific diseases.

File Name: Supplementary Data 5
Description: List of the 57 meconium proteins utilised in the gestational prediction model.
