# Supplementary material for: Host-derived protein profiles of human neonatal meconium across gestational ages
Source: Nat Commun. 2024 Jul 17;15:5543. doi: 10.1038/s41467-024-49805-w (PMC11255260; doi:10.1038/s41467-024-49805-w)
Supplement: Supplementary file 9 — Reporting Summary [file 41467_2024_49805_MOESM9_ESM.pdf]

Corresponding author(s): Eiichiro Watanabe, Yusuke Kawashima

Last updated by author(s): 8 June, 2024

Reporting Summary

Nature Portfolio wishes to improve the reproducibility of the work that we publish. This form provides structure for consistency and transparency in reporting. For further information on Nature Portfolio policies, see our Editorial Policies and the Editorial Policy Checklist.

Statistics

For all statistical analyses, confirm that the following items are present in the figure legend, table legend, main text, or Methods section.

n/a	Confirmed	
☐	☒	The exact sample size (*n*) for each experimental group/condition, given as a discrete number and unit of measurement
☐	☒	A statement on whether measurements were taken from distinct samples or whether the same sample was measured repeatedly
☐	☒	The statistical test(s) used AND whether they are one- or two-sided<br>*Only common tests should be described solely by name; describe more complex techniques in the Methods section.*
☐	☒	A description of all covariates tested
☐	☒	A description of any assumptions or corrections, such as tests of normality and adjustment for multiple comparisons
☐	☒	A full description of the statistical parameters including central tendency (e.g. means) or other basic estimates (e.g. regression coefficient) AND variation (e.g. standard deviation) or associated estimates of uncertainty (e.g. confidence intervals)
☐	☒	For null hypothesis testing, the test statistic (e.g. *F*, *t*, *r*) with confidence intervals, effect sizes, degrees of freedom and *P* value noted<br>*Give P values as exact values whenever suitable.*
☒	☐	For Bayesian analysis, information on the choice of priors and Markov chain Monte Carlo settings
☒	☐	For hierarchical and complex designs, identification of the appropriate level for tests and full reporting of outcomes
☐	☒	Estimates of effect sizes (e.g. Cohen's *d*, Pearson's *r*), indicating how they were calculated

Our web collection on statistics for biologists contains articles on many of the points above.

Software and code

Policy information about availability of computer code

Data collection	Mass spectrometry data were acquired by Xcalibur v4.3 (Thermo Fisher Scientific).
Data analysis	Mass spectrometry files were searched for protein identification using DIA-NN v1.8. R packages clusterProfiler (v4.6.2), fgsea (v1.24.0), glmnet (v4.1.8), and ggplot2 (v3.4.3) were used for analysis and visualization of the proteome data. The codes used in this study are available from the corresponding author upon reasonable request.<br>The code used in this paper can be obtained from GitHub (https://github.com/my0916/meconium).

For manuscripts utilizing custom algorithms or software that are central to the research but not yet described in published literature, software must be made available to editors and reviewers. We strongly encourage code deposition in a community repository (e.g. GitHub). See the Nature Portfolio guidelines for submitting code & software for further information.

Data

Policy information about availability of data

All manuscripts must include a data availability statement. This statement should provide the following information, where applicable:

- Accession codes, unique identifiers, or web links for publicly available datasets
- A description of any restrictions on data availability
- For clinical datasets or third party data, please ensure that the statement adheres to our policy

The MS data of 259 samples and 79 external samples used in this study are available in the ProteomeXchange Consortium via the jPOST partner repository under

the accession codes PXD047426 for ProteomeXchange and JPST002405 for jPOST (https://repository.jpostdb.org/entry/JPST002405.0), and PXD050164 for ProteomeXchange and JPST002961 for jPOST (https://repository.jpostdb.org/entry/JPST002961.0), respectively.

Research involving human participants, their data, or biological material

Policy information about studies with human participants or human data. See also policy information about sex, gender (identity/presentation), and sexual orientation and race, ethnicity and racism.

Reporting on sex and gender	In this study, we exclusively gathered data regarding participants' sex, which is a biological attribute, and consistently referred to it by its accurate terminology. The sex variable was categorized into two groups: Male and Female. Overall, our study encompassed 112 male participants and 147 female participants, with a male-to-female ratio of 43.2% to 56.8% in 259 samples, and 40 male participants and 39 female participants, with a male-to-female ratio of 50.6% to 49.4% in an external validation of 79 samples. 22 males and 18 females, with a male-to- female ratio of 55.0% to 45.0%, contributed another 40 samples of mucin 2 for ELISA analysis. Notably, we did not collect information on gender identity in all three cohorts because the subjects were early postnatal newborns.
Reporting on race, ethnicity, or other socially relevant groupings	The patient characteristics are shown in Table 1 and Table 2, but there is no description regarding race or ethnicity.
Population characteristics	Provided in Table 1, Table 2, and Supplementary Figure 4. 259 newborns, cared in the university of Tokyo hospital were recruited in this prospective study. 20 extremely preterm infants and 23 extremely low birth weight infants were included. Among the total of 259 cases, there were 11 cases with gastrointestinal disease, 42 cases with congenital heart disease, 10 cases with chromosomal abnormality, and 4 cases with congenital infection disease. 79 non-diseased newborns were recruited in the external validation cohort. Among the total of 79 cases, 10 extremely preterm infants and 13 extremely low birth weight infants were included. In 40 samples of mucin 2 for ELISA analysis, 10 extremely preterm infants and 12 extremely low birth weight infants were included.
Recruitment	This prospective study involved the recruitment of newborns receiving care at the University of Tokyo Hospital. Newborns for whom consent from their parents could not be obtained, those from whom initial meconium could not be collected, or those born outside the hospital were excluded from the study.
Ethics oversight	All methods were carried out in accordance with following ethical guidelines in Japan: Ethical Guidelines for Medical and Biological Research Involving Human Subjects. Ethical approval was granted by the Institutional Review Board of the University of Tokyo Hospital (number 2019010NI-13). Additionally, informed consent was obtained from the parents or legal guardians of all participants.

Note that full information on the approval of the study protocol must also be provided in the manuscript.

Field-specific reporting

Please select the one below that is the best fit for your research. If you are not sure, read the appropriate sections before making your selection.

☒ Life sciences ☐ Behavioural & social sciences ☐ Ecological, evolutionary & environmental sciences

For a reference copy of the document with all sections, see nature.com/documents/nr-reporting-summary-flat.pdf

Life sciences study design

All studies must disclose on these points even when the disclosure is negative.

Sample size	No statistical methods were used to pre-determine size of samples. Samples size was planned to be larger than in previous similar reports.
Data exclusions	No data are excluded from any analysis.
Replication	No applicable for this study.
Randomization	Not applicable since this is prospective observational research.
Blinding	Blinding was not applied to this study because this is prospective observational research.

Reporting for specific materials, systems and methods

We require information from authors about some types of materials, experimental systems and methods used in many studies. Here, indicate whether each material, system or method listed is relevant to your study. If you are not sure if a list item applies to your research, read the appropriate section before selecting a response.

Materials & experimental systems

n/a	Involved in the study
☒	☐ Antibodies
☒	☐ Eukaryotic cell lines
☒	☐ Palaeontology and archaeology
☒	☐ Animals and other organisms
☒	☐ Clinical data
☒	☐ Dual use research of concern
☒	☐ Plants

Methods

n/a	Involved in the study
☒	☐ ChIP-seq
☒	☐ Flow cytometry
☒	☐ MRI-based neuroimaging

Plants

Seed stocks	*Report on the source of all seed stocks or other plant material used. If applicable, state the seed stock centre and catalogue number. If plant specimens were collected from the field, describe the collection location, date and sampling procedures.*
Novel plant genotypes	*Describe the methods by which all novel plant genotypes were produced. This includes those generated by transgenic approaches, gene editing, chemical/radiation-based mutagenesis and hybridization. For transgenic lines, describe the transformation method, the number of independent lines analyzed and the generation upon which experiments were performed. For gene-edited lines, describe the editor used, the endogenous sequence targeted for editing, the targeting guide RNA sequence (if applicable) and how the editor was applied.*
Authentication	*Describe any authentication procedures for each seed stock used or novel genotype generated. Describe any experiments used to assess the effect of a mutation and, where applicable, how potential secondary effects (e.g. second site T-DNA insertions, mosiacism, off-target gene editing) were examined.*
